# Detection of interfraction displacement and volume variance during radiotherapy of primary thoracic esophageal cancer based on repeated four-dimensional CT scans

**DOI:** 10.1186/1748-717X-8-224

**Published:** 2013-09-27

**Authors:** Jin Zhi Wang, Jian Bin Li, Wei Wang, Huan Peng Qi, Zhi Fang Ma, Ying Jie Zhang, Ting Yong Fan, Qian Shao, Min Xu

**Affiliations:** 1 Department of Radiation Oncology (Chest Section), Shandong Cancer Hospital, Jinan, Shandong Province 250117, P.R. China; 2 Medicine and life sciences college of Shandong Academy of Medical Sciences, Jinan University, Jinan, Shandong Province 250200, P.R. China; 3 Department of Radiation Oncology, Tai’an Tumor Prevention and Treatment Hospital, Tai’an, Shandong Province 271000, P.R. China

**Keywords:** Esophageal cancer, Radiotherapy, 4DCT, Primary tumor volume, Interfractional displacement

## Abstract

**Background:**

To investigate the interfraction displacement and volume variation of primary thoracic esophagus carcinoma with enhanced four-dimensional computed tomography (4DCT) scanning during fractionated radiotherapy.

**Methods:**

4DCT data sets were acquired at the time of treatment simulation and every ten fraction for each of 32 patients throughout treatment. Scans were registered to baseline (simulation) 4DCT scans by using bony landmarks. The gross tumor volumes (GTVs) were delineated on each data set. Coordinates of the GTV centroids were acquired on each respiration phase. Distance between center of the GTV contour on the simulation scan and the centers on subsequent scans were used to assess interfraction displacement between fractions. Volumes were constructed using three approaches: The GTV delineated from the maximum intensity projection (MIP) was defined IGTV_MIP_, all 10 GTVs were combined to form IGTV_10_, GTV_mean_ was the average of all 10 phases of each GTV.

**Results:**

Interfraction displacement in left-right (LR), anterior-posterior (AP), superior-inferior (SI) directions and 3D vector were 0.13 ± 0.09 cm, 0.16 ± 0.12 cm, 0.34 ± 0.26 cm and 0.43 ± 0.24 cm, respectively between the tenth fraction and simulation 4DCT scan. 0.14 ± 0.09 cm, 0.19 ± 0.16 cm, 0.45 ± 0.43 cm and 0.56 ± 0.40 cm in LR, AP, SI and 3D vector respectively between the twentieth fraction and simulation 4DCT scan. Displacement in SI direction was larger than LR and AP directions during treatment. For distal esophageal cancer, increased interfraction displacements were observed in SI direction and 3D vector (P = 0.002 and P = 0.001, respectively) during radiotherapy. The volume of GTV_mean_, IGTV_MIP_, and IGTV_10_ decreased significantly at the twentieth fraction for middle (median: 34.01%, 33.09% and 28.71%, respectively) and distal (median: 22.76%, 25.27% and 23.96%, respectively) esophageal cancer, but for the upper third, no significant variation were observed during radiotherapy.

**Conclusions:**

Interfractional displacements in SI direction were larger than LR and AP directions. For distal location, significant changes were observed in SI direction and 3D vector during radiotherapy. For middle and distal locations, the best time to reset position should be selected at the twentieth fraction when the primary tumor target volume changed significantly, and it was preferable to guide target correction and planning modification.

## Background

Radiotherapy (RT) plays an important role in the treatment of esophageal cancer, the three-dimensional conformal radiotherapy (3D-CRT) and intensity-modulated radiotherapy (IMRT) are the most important delivery platforms [1,2]. Precise definition of RT fields is crucial for RT planning. Variation of target volume and displacement are the sources for RT fields and plan modification, such changes can be intrafractional or interfractional. Intrafractional esophageal motion can be attributed mostly to respiration, cardiac activity, and esophageal peristalsis [3], which has been well documented [4-8]. But studies about interfractional esophageal motion were limited. As a consequence of radiation treatment, tumor volumes will change during radiotherapy, significant regression in lung tumor volume can occur by 3 weeks after beginning treatment [9-11]. But so far, no conclusive data exist as to the nature of the tumor volume changes during radiotherapy for primary esophageal cancer, or the time at which these changes occur.

Relative to three-dimensional computed tomography (3DCT), four-dimensional computed tomography (4DCT) scan could not only obtain the volume of primary tumor GTV without motion information, for example, the GTV delineated on a single phase; but also obtain internal gross tumor volume (IGTV) volume with entire motion information, for example, the IGTV combined from 10 phases. In addition, we also can obtain IGTV_MIP_ from the maximum intensity projection (MIP). Therefore, based on repeated 4DCT, we can obtain more precise variation of target volume during entire treatment for primary esophageal cancer. In present study, we measured the interfractional displacement of the GTV, and variation of GTV/IGTV in conventional fractioned RT during treatment for primary esophageal cancer using repeated 4DCT.

## Methods

### Patient characteristics

A total of 32 patients with pathologically confirmed thoracic esophageal cancer were considered eligible for radiotherapy with 3DCRT or IMRT from August 2011 to October 2012. 32 patients completed the simulation 4DCT scan and the tenth fractional scan, 27 patients completed the twentieth fractional scan. Patients with poor pulmonary function or preexisting respiratory problems were excluded. Written informed consent was obtained from all of the patients before the treatment was initiated. The patient characteristics are listed in Table 1.

**Table 1:** Patient characteristics

**Characteristic**	**Number**
Sex, n (%)	
M	26 (81.3%)
F	6 (18.7%)
Age, median, y (range)	71 (45–89)
Tumor histology, n (%)	
Squamous cell carcinoma	25 (78.1%)
Adenocarcinoma	7 (19.9%)
Disease stage, n (%)	
I	6 (18.8%)
II	12 (37.5%)
III	5 (15.6%)
IV	9 (28.1%)
Tumor location, n (%)	
Upper	9 (28.1%)
Mid-	14 (43.8%)
Distal	9 (28.1%)

### CT data acquisition

Every patient underwent a 4DCT scan on a 16-slice CT scanner (Philips Brilliance Bores CT, Netherlands). All of the patients were scanned in supine position with arms stretched over the head using the vacuum bag, followed by laser alignment. Metal marks were applied to the laser cross marked points in the bilateral axial midline and the anterior midline. Images were obtained from the neck to the mid-abdomen using the axial CT mode, and all these scans were gathered during free breathing (FB) without any breathing control. During the 4DCT image acquisition, the patient’s respiration was monitored using the Real-Time Position Management (RPM) Respiratory Gating System (Varian Medical Systems, Palo Alto, CA) by tracking the trajectory of the infrared markers placed on the patient’s abdomen. The signal was sent to the scanner to label a time tag on each CT image. GE Advantage 4D software (GE Healthcare, Waukesha, WI) sorts the reconstructed 4DCT images into ten respiratory phases labeled as 0% - 90% on the basis of these tags, with 0% corresponding to end inspiration (EI) and 50% corresponding to end expiration (EE). The 4DCT images were reconstructed using a thickness of 3 mm and then transferred to the Eclipse treatment planning system (TPS) (Eclipse 8.6, Varian Medical Systems, Palo Alto, CA) for structure delineation and treatment planning generation.

### GTV delineation, volume and displacement determination

All of the 4DCT data sets of each patient were registered to the reference 4DCT scanning (the first 4DCT scan/simulation) corresponding to the end expiration phase (respiratory phase 50%, GTV_50_) using software tools in the radiation treatment-planning system on the basis of bony landmarks for comparison. For each 4DCT data set, the primary tumor (was considered as the GTV) was drawn by a single physician with same window level and window widths. The full respiration GTV centroid positions were acquired by the Varian Eclipse 8.6 treatment planning system. Volumes were constructed using three approaches: GTV_mean_ was the average of all 10 phases of each GTV, IGTV_MIP_ was the contour delineated from the MIP, all 10 GTVs were combined to form IGTV_10_. Displacement in each direction between the center of the GTV_50_ contour on the simulation scan and the centers on subsequent scans was used to assess interfraction displacement between fractions, which was obtained by coordinates of the GTV_50_ centroid on the subsequent datasets subtracted that on the reference dataset. In addition, the three dimensional tumor motion vector was obtained using motion data in the different axes.

### Statistical analysis

The displacement among three directions and the displacement in the same direction among different locations during the same fraction were used by one-way ANOVA. The displacements on the same direction among all fractions and volumes among all fractions were used by a paired sample T test. Values of P < 0.05 were considered significant. All statistical analyses were performed using the SPSS software package.

## Results

### Comparison of the interfraction displacement for the tenth fraction 4DCT scan

For all of the patients, slightly larger displacements were observed in the SI direction with mean ± standard deviation (SD) of 0.34 ± 0.26 cm, compared with 0.13 ± 0.09 cm (P = 0.000) and 0.16 ± 0.12 cm (P = 0.001) in the LR and AP directions, respectively. The mean magnitude of the interfractional GTV centroid positional variations for the upper, middle, and distal esophageal cancer were summarized in Table 2. The displacements in the SI direction were also larger than LR and AP directions (P = 0.024, P = 0.028; P = 0.049, 0.047; P = 0.000, P = 0.001; respectively). The displacements in the distal location were larger than in the upper and middle tumor locations in the SI direction and the 3D vector.

**Table 2:** Magnitude (cm) of interfraction GTV centroid motion for the tenth fraction 4DCT scan

**Group**	**LR**			**AP**			**SI**			**F**	**P**	**3D vector**		
	**Range**	**Mean**	**SD**	**Range**	**Mean**	**SD**	**Range**	**Mean**	**SD**			**Range**	**Mean**	**SD**
Upper	0.01-0.35	0.13	0.11	0.01-0.26	0.13	0.09	0.01-0.65	0.31	0.25	3.745	0.038	0.03-0.78	0.39	0.23
Mid-	0.02-0.32	0.14	0.10		0.01-0.46	0.15	0.13	0.01-0.75	0.33	0.23	3.806	0.034	0.05-0.77	0.41	0.23
Distal	0.01-0.17	0.14	0.04	0.08-0.50	0.21	0.13	0.13-0.87	0.51	0.25	13.672	0.000	0.25-0.89	0.59	0.22	
F	0.168			0.892			3.681					3.251			
P	0.846			0.421			0.038					0.048			

*Abbreviation*: *GTV* gross tumor volume, *4DCT* four-dimensional computed tomography, *LR* right–left, *AP* anterior–posterior, *SI* superior–inferior, *3D* three-dimensional, *SD* standard deviation; “F”, the ratio of mean square about “between groups” and “within groups” used by one-way ANOVA; “P” , the significance based on two-tailed p values. Values of P < 0.05 were considered significant.

### Comparison of the interfraction displacement for the twentieth fraction 4DCT scan

For all of the patients, displacement in LR, AP, and SI direction were 0.14 ± 0.09 cm, 0.19 ± 0.16 cm, and 0.45 ± 0.43 cm, respectively. The displacement in SI direction was larger than LR (P = 0.001) and AP (P = 0.007) direction. The mean magnitude of the interfractional GTV centroid positional variations for upper, middle, and distal esophageal cancer were summarized in Table 3. The displacements in SI direction were also larger than in LR and AP directions (P = 0.011, P = 0.028; P = 0.048, 0.045; P = 0.000, P = 0.000; respectively). The displacements in the distal location were larger than in the upper and middle location in the AP, SI direction and the 3D vector.

**Table 3:** Magnitude (cm) of interfraction GTV centroid motion for the twentieth fraction 4DCT scan

**Group**	**LR**			**AP**			**SI**			**F**	**P**	**3D vector**		
	**Range**	**Mean**	**SD**	**Range**	**Mean**	**SD**	**Range**	**Mean**	**SD**			**Range**	**Mean**	**SD**
Upper	0.04-0.14	0.08	0.04	0.01-0.39	0.13	0.13	0.09-1.30	0.40	0.39	4.405	0.023	0.12-1.30	0.46	0.38
Mid-	0.02-0.34	0.14	0.10	0.01-0.41	0.16	0.14	0.01-0.55	0.37	0.20	3.614	0.041	0.02-0.58	0.42	0.17
Distal	0.01-0.54	0.16	0.10	0.01-0.54	0.31	0.16	0.45-1.66	0.86	0.39	17.919	0.000	0.54-1.67	0.96	0.36
F	3.132			3.565			10.055					9.318		
P	0.062			0.044			0.001					0.001		

*Abbreviation*: *GTV* gross tumor volume, *4DCT* four-dimensional computed tomography, *LR* right–left, *AP* anterior–posterior, *SI* superior–inferior, *3D* three-dimensional, *SD* standard deviation. “F”, the ratio of mean square about “between groups” and “within groups” used by one-way ANOVA; “P” , the significance based on two-tailed p values. Values of P < 0.05 were considered significant.

### Changes of interfraction displacement of the GTV centroid during treatment

For the upper and middle tumor locations, no significant differences were found in each direction and the 3D vector. For the distal esophageal cancer, significant differences were observed in SI direction and the 3D vector (P = 0.002 and P = 0.001, respectively) during radiotherapy.

### Variation of tumor volumes

**Figure 1:**
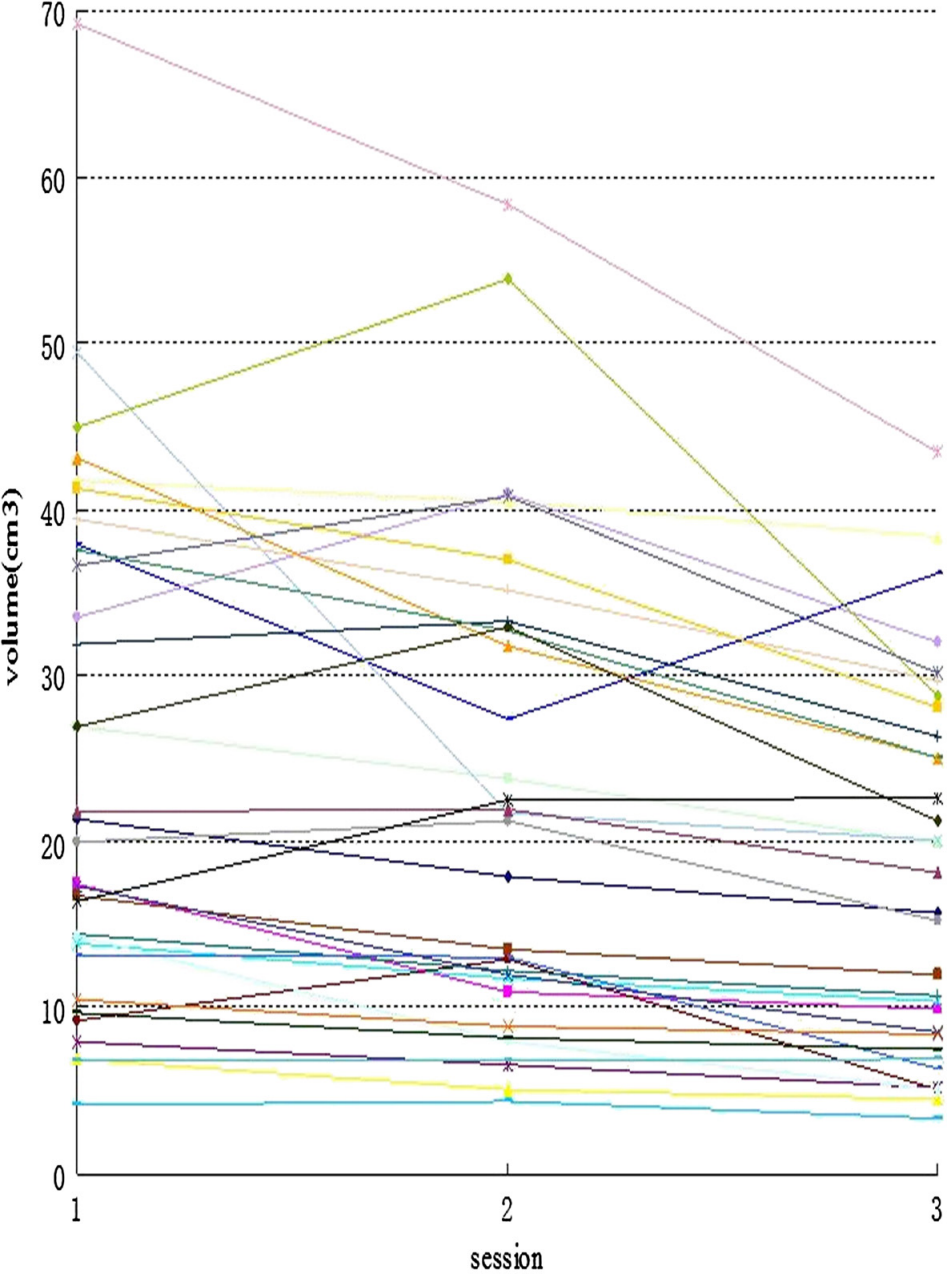
**Absolute volumetric changes of GTV**_**mean **_**during 3DCRT/IMRT (n = 32).**

The tumor volumes variation of the GTV_mean_, the IGTV_MIP_ and the IGTV_10_ during radiotherapy are listed in Table 4. The tumor volume showed a trend of decline during the entire treatment, the change trend of GTV_mean_, IGTV_MIP_, and IGTV_10_ are completely consistent. At the tenth fraction, the volume of GTV_mean_, IGTV_MIP_, and IGTV_10_ decreased, but the variations are not significant compared to primary volumes for the upper, middle and distal tumor locations. For the GTV_mean_, volumes are increased 22.41% in 7 of 32 patients (range, 4.20%-39.42%), differences are statistically significant (P = 0.003). At the twentieth fraction, tumor volumes shrink further more, for all of the tumors, the volume of GTV_mean_, IGTV_MIP_, and IGTV_10_ decreased significantly relative to those of primary volumes (P = 0.000, P = 0.000 and P = 0.000, respectively). For the middle location, the GTV_mean_ volume decreased 34.01% (range, 13.45%-63.28%, P = 0.009), IGTV_MIP_ 33.09% (range, 6.00%-58.00%, P = 0.007), IGTV_10_ 28.71% (range, 2.00%-54.00%, P = 0.012). For the distal esophageal cancer, GTV_mean_ volume decreased 22.76% (range, 14.01%-50.64%, P = 0.047), IGTV_MIP_ 25.27% (range, 9.00%-48.00%, P = 0.024), IGTV_10_ 23.96% (range, 11.00%-51.00%, P = 0.029). But for the upper location, no significant reduction to the volume of GTV_mean_, IGTV_MIP_, and IGTV_10_ (P = 0.079, P = 0.082, and P = 0.164, respectively). Figure 1 showed the absolute volumetric changes of the GTV_mean_ during 3DCRT/IMRT.

**Table 4:** Variation of tumor volume during radiotherapy(cm3,mean ± SD,)

**Group**	**GTV**_**mean**_			**IGTV**_**MIP**_			**IGTV**_**10**_		
	**Simulation**	**Tenth**	**Twentieth**	**Simulation**	**Tenth**	**Twentieth**	**Simulation**	**Tenth**	**Twentieth**
Upper	14.82 ± 10.22	12.15 ± 7.15	11.24 ± 10.17	15.5 ± 10.00	12.84 ± 7.61	11.84 ± 10.37	19.95 ± 14.30	16.44 ± 10.44	16.89 ± 18.41
Mid-	33.63 ± 17.15	29.63 ± 17.39	24.21 ± 13.25	35.18 ± 18.29	31.16 ± 18.22	25.33 ± 13.90	42.40 ± 21.18	37.86 ± 21.46	31.69 ± 16.49
Distal	22.04 ± 8.67	20.92 ± 8.69	17.69 ± 7.43	23.10 ± 8.67	21.96 ± 9.18	18.70 ± 7.81	29.68 ± 11.09	27.90 ± 12.57	23.98 ± 10.40
Total	25.08 ± 15.53	22.35 ± 14.59	17.95 ± 11.75	26.25 ± 16.07	23.62 ± 15.33	18.87 ± 12.23	32.51 ± 19.10	28.88 ± 18.30	24.47 ± 16.36

*Abbreviation*: *GTV* gross tumor volume, *IGTV* internal gross tumor volume, *MIP* maximum intensity projection, *simulation* the simulation 4DCT scan, *tenth* the tenth 4DCT scan, *twentieth* the twentieth 4DCT scan.

## Discussion

The accurate definition of a target is crucial for the delivery of high-precision radiotherapy in esophageal cancer. The planning target volume (PTV) is defined as the clinical target volume plus an internal margin (IM) that includes the target internal motion and daily setup error (SM) [12]. Some researchers have reported that the tumor position varies intrafractionally, but interfractional displacement and tumor volume variation during radiotherapy were limited. The aim of this study was to introduce interfraction displacement and regression of tumor volume over entire treatment for primary esophageal cancer.

Interfractional displacement is defined as displacement of the tumor position relative to its position at simulation between fractions, reports of which have been limited. In this study, we analyzed interfraction displacement not only in whole esophagus but also in upper, middle, and distal esophagus. In addition, we compared the displacement on same direction among different locations, and the displacement on same direction over entire treatment.

In the present study, interfractional displacement in the SI direction is larger than LR and AP direction whether for whole esophagus or different locations during entire course of treatment (Table 2 and Table 3). These results are consistent with the reported data from Wang et al. [13], who used 4DCT to analyze interfractional displacement for 22 esophageal malignancy patients at the end expiration phase. They demonstrated that the interfractional displacement of the gastroesophageal junction (GEJ) in the SI, AP, and LR directions were 6.77 mm (maximum displacement, 17.6 mm), 2.90 mm and 2.88 mm, respectively. They also confirmed that the interfractional systematic displacement in the SI direction correlated strongly with the interfractional change in tidal volume (r = 0.9635) and vertical diaphragmatic displacement (r =0.9437). Perhaps this is the reason why displacement in the SI direction is larger than in the LR and AP direction. Cohen et al. [14] used CT-on-rails to study 8 patients with esophageal tumors and found a mean absolute esophageal displacement of 3.2 mm below and 4.2 mm above the carina in the LR direction and a mean absolute AP displacement of 2.8 mm posterior below and 3.9 mm posterior above the carina. Another study [15] using cone-beam CT to analyze esophageal shifts in 20 patients with esophageal cancer showed similar 5 mm in the LR direction and 5 mm in the AP direction. However, the two studies above did not address interfractional esophageal motion in the SI direction. The study from Wang et al. [13] did include the SI direction, but they limited their study to the GEJ and did not divide the esophagus into different segments.

In our study, we found displacement in the SI direction and 3D vector in the distal location were larger than upper and middle third esophagus during radiotherapy. This finding suggests that displacement in the distal esophagus is large, especially in the SI direction. Wang’s study [13] also found large interfractional SI displacements. We also analyzed displacement variation on the same direction over entire treatment. For the upper and middle locations, no significant differences were found in each direction and 3D vector. For the distal esophageal cancer, significant differences were observed in the SI direction and 3D vector (P = 0.002 and P = 0.001, respectively) during radiotherapy. Along with treatment, the displacement in SI direction (0.86 ± 0.39 cm vs 0.51 ± 0.25 cm) and 3D vector (0.96 ± 0.36 cm vs 0.59 ± 0.22 cm) for distal patients increased. Suggesting that expanding margin reasonably in SI direction is needed for the distal patients at the twentieth fraction. Wang et al. [13] analyzed one patient who had the largest inferior systematic displacement; the CTV was underdosed, which resulted in higher-than-expected doses to the GEJ, and these hot spots also affected exposure to the normal stomach and lung. Their findings thus justified at least a 10 mm inferior PTV margin. This reminds us that we must pay great attention to interfractional displacement at the twentieth fraction, particularly for distal patients, to prevent a high radiation dose to the normal tissues and an insufficient dose to the target.

Monitoring the regression and deformation for tumor volume could help revise the target and treatment plan in time. In this study, we found the majority of the tumor volumes were decreased with increasing fractions during radiotherapy based on repeated 4DCT scanning. However, at the tenth fraction, the volume of the GTV_mean_ increased 22.41% in 7 of 32 patients (range, 4.20%-39.42%). Similar to our study, Britton et al. [10] used 4DCT to assess GTV regression during radiotherapy for non-small-cell lung cancer; they showed a transient increased in the GTV volume for 5 patients during the first or second week of treatment. The case was also observed in intracranial lesions [16-22]. In the process of sketch, we found that the tumor and esophageal mucosa nearby thickened locally, but by only relying on CT imaging, we cannot judge whether the increase is caused by oedema or infiltration. Therefore, according to the studies above, clinicians should pay attention to tumor volume change at the initial treatment, because target mispositioning will result in a high radiation dose to the normal tissues and an insufficient dose to the target.

Along with the progress of radiotherapy treatment, the tumor size decreased significantly. In our study, except for upper third esophagus, the GTV_mean_, IGTV_MIP_, and IGTV_10_ of middle and distal esophageal cancer decreased significantly at the twentieth fraction. Studies on lung cancer concluded that significant changes in the target volume occurred after 2 weeks. For example, A study from Underberg et al. [23], who identify potential time trends in target volumes and tumor mobility after stereotactic radiotherapy (SRT) for Stage I non–small-cell lung cancer in 40 tumors, showed that GTV and ITV decreased significantly at the fourth week after the start of treatment (P < 0.015). In our study, we found the larger the absolute initial tumor volume, the greater the absolute tumor volume shrinking. In the present study, the tumor volume in the upper third is smaller than that of mid- and distal segment (P = 0.003), perhaps this is the reason to the differentiation of volume change in different locations. Our study showed that the tumor volume in 2 patients increased continuously over entire course of treatment. We analyzed the probable causes may be related to image artifacts affecting the numbers of slices contoured. We could not rule out the cause of the tumor growth.

In addition, a similar time trend as for the GTV_mean_ was observed for the IGTV_10_ and IGTV_MIP_, with an overall decrease during radiotherapy. Just as the GTV_mean_, the volumes also decreased significantly at the twentieth fraction. IGTV_10_ was fused by GTVs on ten phases, contained mobility information of entire respiratory cycle [24,25]. Our results show that the GTV or IGTV volume changes significantly at the twentieth fraction of treatment, suggesting the need for reimaging and potential replanning for some patients.

## Conclusions

We investigated the interfraction displacement and tumor volume variation during radiotherapy for primary esophageal cancer. Larger displacement in SI direction was observed, significant variation were exist in SI direction and 3D vector for distal esophageal cancer during radiotherapy. Tumor volume decreased significantly at the twentieth fraction. Therefore, for primary middle and distal esophageal cancer, the best time to reset the position may be at the twentieth fraction when the primary tumor target volume changed significantly. Resetting the position is recommended to guide the target correction and treatment planning modification.

## Abbreviations

4DCT: Four-dimensional computed tomography; IGTV: Internal gross tumor volume; MIP: Maximum intensity projection; LR: Left-right; AP: Anterior-posterior; SI: Superior-inferior; PTV: Planning target volume; RPM: Real time positioning management; TPS: Treatment planning system; SD: Standard deviation; 3D-CRT: Three-dimensional conformal radiotherapy; IMRT: Intensity-modulated radiotherapy.

## Competing interests

The authors declare that they have no competing interests.

## Authors’ contributions

JZW, JBL participated in the study design, contributed to the data collection, and draft the manuscript. WW, HPQ, ZFM and YJZ made important contributions in the design of the study and in revising the content. TYF, QS and MX contributed in collecting and analyzing data. All authors read and approved the final manuscript.
